# Mobile applications in adolescent psychotherapy during the COVID-19 pandemic: a systematic review

**DOI:** 10.3389/fpubh.2024.1345808

**Published:** 2024-02-14

**Authors:** Sarah Wüllner, Katharin Hermenau, Mariya Krutkova, Ira-Katharina Petras, Tobias Hecker, Michael Siniatchkin

**Affiliations:** ^1^University Clinic of Child and Adolescent Psychiatry and Psychotherapy, Protestant Hospital Bethel, Bielefeld University, Medical School East Westphalia, Bielefeld, Germany; ^2^Department of Psychiatry, Psychotherapy and Psychosomatics of Children and Adolescents, University Hospital Rheinisch-Westfälische Technische Hochschule Aachen, Aachen, Germany; ^3^Institute for Interdisciplinary Conflict and Violence Research, Bielefeld University, Bielefeld, Germany; ^4^Department of Psychology, Faculty of Psychology and Sports Science, Bielefeld University, Bielefeld, Germany

**Keywords:** mental health, app, adolescent, youth, psychotherapy, mHealth, feasibility, effectiveness

## Abstract

**Background:**

To bridge the gap in adolescent psychotherapy created by the increasing need for mental health interventions and the limited possibilities of in-person treatment during the pandemic, many health care providers opted to offer online mental health care programs. As a result, the number of mental health apps available in app stores experienced a sharp increase during the COVID-19 pandemic.

**Objective:**

The aim of the current review is to provide an overview of feasibility and effectiveness studies testing mobile applications in adolescent psychotherapy during the peak phase of the COVID-19 pandemic.

**Methods:**

We conducted a literature search in Pubmed, PsychInfo, Google Scholar, OpenSIGLE and OpenGREY for papers published from June 2020 to June 2023. Studies were included if they evaluated app-based interventions intended for psychotherapeutic treatment and targeted adolescents between 12 and 27 years of age with symptoms of psychological disorders. The quality of each study was assessed using the Systematic Assessment of Quality in Observational Research (SAQOR). Effectiveness outcomes were analyzed by vote counting and calculating a binomial probability test.

**Results:**

The search yielded 31 relevant studies that examined 27 different apps with a total of 1,578 adolescent participants. Nine articles were primary effectiveness studies and 22 focused on feasibility measures as primary outcome. There was evidence that mental health apps influenced adolescents' psychotherapy, with 83% of the studies with effectiveness outcomes favoring the intervention (*p* = 0.002). Sixty-one percent of the included studies were rated at low or very low quality.

**Conclusions:**

The pandemic has given apps a firm and important role in healthcare that will probably continue to expand in the future. To ensure that mental health apps are truly effective and beneficial for adolescents' psychotherapy, we need a standardized measurement of quality features of mental health apps and higher quality app evaluation studies.

**Systematic review registration:**

https://www.crd.york.ac.uk/PROSPERO/display_record.php?RecordID=406455, PROSPERO International Prospective Register of Systematic Reviews [CRD42023406455].

## 1 Introduction

Due to the Coronavirus Disease 2019 (COVID-19) pandemic mental health problems increased dramatically, especially among adolescents (1–7). Additionally, our health care system experienced rapid digitalization. Many organizations developed mobile applications to maintain their treatment offers under the conditions of social distancing (8, 9). This resulted in a sharp increase of available mental Health apps (10, 11) as well as published evaluation studies (9). Most of the published studies and reviews of evaluated mental health apps focus on the adult population. Research on app-based interventions specifically for adolescents is still scarce. Nevertheless, Ellis et al. (9) reported that children and adolescents were identified as one of the most frequently targeted specific populations in published app evaluation studies during the pandemic. Comparing the number of mental health apps available in app stores and published app evaluation studies, a high discrepancy can be found (9–11). The majority of available mental health apps failed to demonstrate their effectiveness (12, 13). However, evaluation studies are an important quality feature. Without evaluation studies it is difficult to determine whether mental health apps are truly beneficial or potentially harmful for the mental health of users (14, 15). Previous research found that several non-evaluated mental health apps provided incorrect psychoeducation information, inappropriate treatment strategies or wrong contact details of emergency services (16, 17). Given the importance of evaluated mental health apps, as well as the greater focus on app-based psychotherapeutic treatment options for adolescents, there is an urgent need for an updated review of evaluation studies of mental health apps in the context of adolescent psychotherapy during the COVID-19 pandemic.

### 1.1 Challenges for psychotherapeutic care during the COVID-19 pandemic

In Germany, Ravens-Sieberer et al. (18) reported an increase of overall mental health problems in adolescents from 18 to 28%. The most common disorders were anxiety disorders and depression, which is comparable to data before the pandemic (18). Studies from other countries found an increase in self-harm, suicidal ideation and attempted suicide (5, 19). Madigan et al. (5) showed in their review that emergency department visits due to self-harm, suicidal ideation or suicidal attempts increased in the beginning of the pandemic despite a reduction in total emergency department visits for mental-illness concerns. In times of social distancing and school closures, adolescents lost important resources for their wellbeing, resulting in negative consequences for their mental health (7, 20, 21). Additionally, most of the treatment services were curtailed or completely discontinued during the phases of social distancing (7). As a result, the youth was exposed to greater stressors during the pandemic, while less support was available. In Germany, we can see the consequences of this in a significant increase of emergency admissions since 2021, especially in child and adolescent psychiatry (22). To address the divergence between the increasing need for mental health treatments and the discontinuation of treatment offers in times of the pandemic, health care providers tried to find new ways to reach adolescents, including offering online mental health care programs (8, 9).

### 1.2 Chances of mental health apps in adolescent psychotherapy

The use of mental health apps with adolescents is promising. Digital media are an integral part of adolescent everyday lives. In Germany, 96% of 12–19-year-olds own a smartphone and use it daily (23). In 2022, adolescents spent on average 204 min per day on the internet. During the pandemic the online usage times were significantly elevated, averaging 244–258 min per day (23). Furthermore, younger people show greater affinity for online mental health care. They are more likely to use the internet to gather information about their mental health than older people (24, 25). Rauschenberg et al. (26) pointed out that a large proportion of young people with psychological distress and pandemic related anxiety would like to use mobile applications to overcome negative consequences of the COVID-19 pandemic. In addition to permanent availability, mental health apps have further advantages, such as allowing adolescents to have more autonomy. They can use apps flexibly and decide when and where to get involved with the app without having to go to a fixed treatment appointment as one would in case of face-to-face therapy (27, 28). Accessibility is one of the most important arguments for using mental health interventions when it comes to adolescents. Therefore, smartphone-based interventions are more attractive to them than interventions where a laptop or other digital device is needed (27). Furthermore, apps can offer immediate support in critical situations and crises, like acute cases of suicidal ideation or self-harm (29). Due to increased affordability of mental health care through apps, it is possible to reach a higher number of help-seeking adolescents. Access to mental health apps is given independent of the available health care infrastructure or severity of symptoms. As such, persons with low to moderate symptoms of mental health conditions can be treated to prevent the development of more severe symptomatology (30). In addition, adolescents perceive the use of mental health apps as less conspicuous and bulky, which may lead to increased adherence to psychotherapy. Feeling of connectedness is also an important factor for adolescents and a further advantage of mental health apps. Mental health apps can offer an opportunity to share own experiences with peers in an appropriate manner and mitigate the fear of stigmatization (27, 28). Finally, considering previous effectiveness research on mental health apps, several studies show comparable efficacy and cost-effectiveness between smartphone-based interventions and face-to-face therapy (26, 31–33).

### 1.3 Areas of application of mental health apps

Just as versatile as the reasons for app usage are their areas of application. Apps can be used as stand-alone or therapy-accompanying treatments. As a stand-alone treatment, apps offer interventions for self-help. For example, they can be used during waiting periods for psychotherapy or as early interventions to prevent the development of severe symptomatology (29, 30, 34–36). Therapy-accompanying apps are used as adjunction to psychotherapy (29). These apps can support adolescents between outpatient sessions. They can increase adherence to therapeutic homework, support application of skills acquired in therapy to everyday life or offer management plans for acute crises (34, 35). Most of the available apps focus on specific disorders rather than a transdiagnostic therapy approach covering the eight common disorders: psychosis, eating disorders, depression, autism, self-harm, anxiety, substance abuse, and suicidal behavior (35). Lui et al. (34) reported in their review of evidence-based mobile applications in a psychotherapy context that none of the 21 included apps focused on symptoms that may be transdiagnostic. Four years later, Ellis et al. (9) reported that during the COVID-19 pandemic the transdiagnostic approach increased in mental health app literature: they found that 38% of the included studies were about COVID-19-related transdiagnostic symptoms like stress, loneliness or general wellbeing.

### 1.4 Evaluation of mental health apps

As mentioned above, there is a large discrepancy between available mental health apps and published evaluation studies of mental health apps. In the first quarter 2021, 53,979 mental health apps were available in Apple App Store and 53,054 in Google Play Store (10, 11). In contrast, Ellis et al. (9) reported in their review that between January 2020 and March 2021 356 app evaluation articles were published, with 63% of these being non-empirical publication types like commentaries or opinions. In another review, Alyami et al. (12) pointed out that none of the 1,154 identified social anxiety apps for adults had published studies of their effectiveness. Three years later, Qu et al. (13) presented in their review that of 482 investigated depression apps for adults only seven percent had a sound evidence base. Other reviews of evidence-based mental health apps for adults highlighted an insufficient scientific evaluation of app-based interventions and a lack of standardized methods for assessing effectiveness of mental health apps (15, 33, 34, 37). One reason for the low rates of effectiveness studies is attributed to the high costs involved. Effectiveness studies require a great deal of effort and usually result in long study periods, which does not meet the requirements of the fast-moving app market (14, 38). Another reason for the high rates of non-evaluated mental health apps is that providers of health care apps are not required to provide information in the app stores about the effectiveness of their digital therapeutic tools (39, 40). Most providers still do not hesitate to claim effectiveness of their applications by means of non-empirical scientific explanations, field reports or technical expertise. If an evaluation of a mental health app is available, it is mostly an evaluation of feasibility (41). Feasibility is an important aspect of an overall assessment of interventions, but it does not provide information about the usefulness or effectiveness of mental health apps. To give a comprehensive overview it is important to include various kinds of evaluation studies, from feasibility to effectiveness.

### 1.5 Objectives

The aim of the current review is to provide an overview of feasibility and effectiveness studies testing mobile applications in adolescent psychotherapy during the COVID-19 pandemic. Furthermore, we investigate how effectiveness of mobile applications is measured. Additionally, we examine whether effects of mobile applications differ according to specific psychological disorders as well as between stand-alone psychotherapy apps and therapy-accompanying apps.

## 2 Methods

A protocol for reviewing the literature was developed using the Preferred Reporting Items for Systematic Reviews and Meta-Analyses (PRISMA) guidelines (42). The review was registered on PROSPERO (CRD42023406455).

### 2.1 Search strategy

A literature search was conducted in Pubmed, PsychInfo and Google Scholar for papers published from June 2020 to June 2023. Search parameters consisted of numerous combinations of keywords related to adolescents, apps and psychotherapy and included “adolescent^*^,” “youth,” “young,” “app,” “mobile,” “smartphone,” “mental health,” “digital,” “psychotherapy,” “disorder,” “psychological,” “psychiatry,” “treatment,” “therapy,” and “intervention.” For eligible gray literature, we searched OpenSIGLE and OpenGREY. Furthermore, authors of study protocols were contacted to check for recently published studies or preliminary study results. References of reviews, meta-analyses, review protocols and included studies were scanned to identify any potentially relevant literature.

### 2.2 Eligibility criteria

Studies were included if they evaluated app-based interventions intended for psychotherapeutic treatment and targeted adolescents between 12 and 27 years of age with symptoms of psychological disorders. Studies addressing smoking were excluded because smoking is not a clinically relevant and psychiatrically or psychotherapeutically treated addictive disease. In addition, studies with only a subset of eligible participants were excluded if it was not possible to consider the subsample separately. Studies exclusively examining adults 18 years of age or older were likewise excluded. We included any mobile app-based intervention in a psychotherapy context for adolescents. The app had to be used as a supplement to or replacement of psychotherapy. It could focus on specific psychological disorders or transdiagnostic treatment. Solely psychoeducational or diagnostic mobile applications were excluded. We included all published, unpublished, or ongoing experimental and quasi-experimental trials in English and German that compared mobile applications in a psychotherapy context with usual psychotherapy or non-psychological mobile applications (e.g., gaming applications); non-experimental studies with repeated measurements design that included at least pre- and post-measurement; and non-experimental studies based on qualitative research methods. Trials described in Editorials, Comments or Letters to the editor were excluded. Due to the COVID-19 pandemic, only studies published from June 2020 to June 2023 were included.

### 2.3 Study selection process

**Figure 1:**
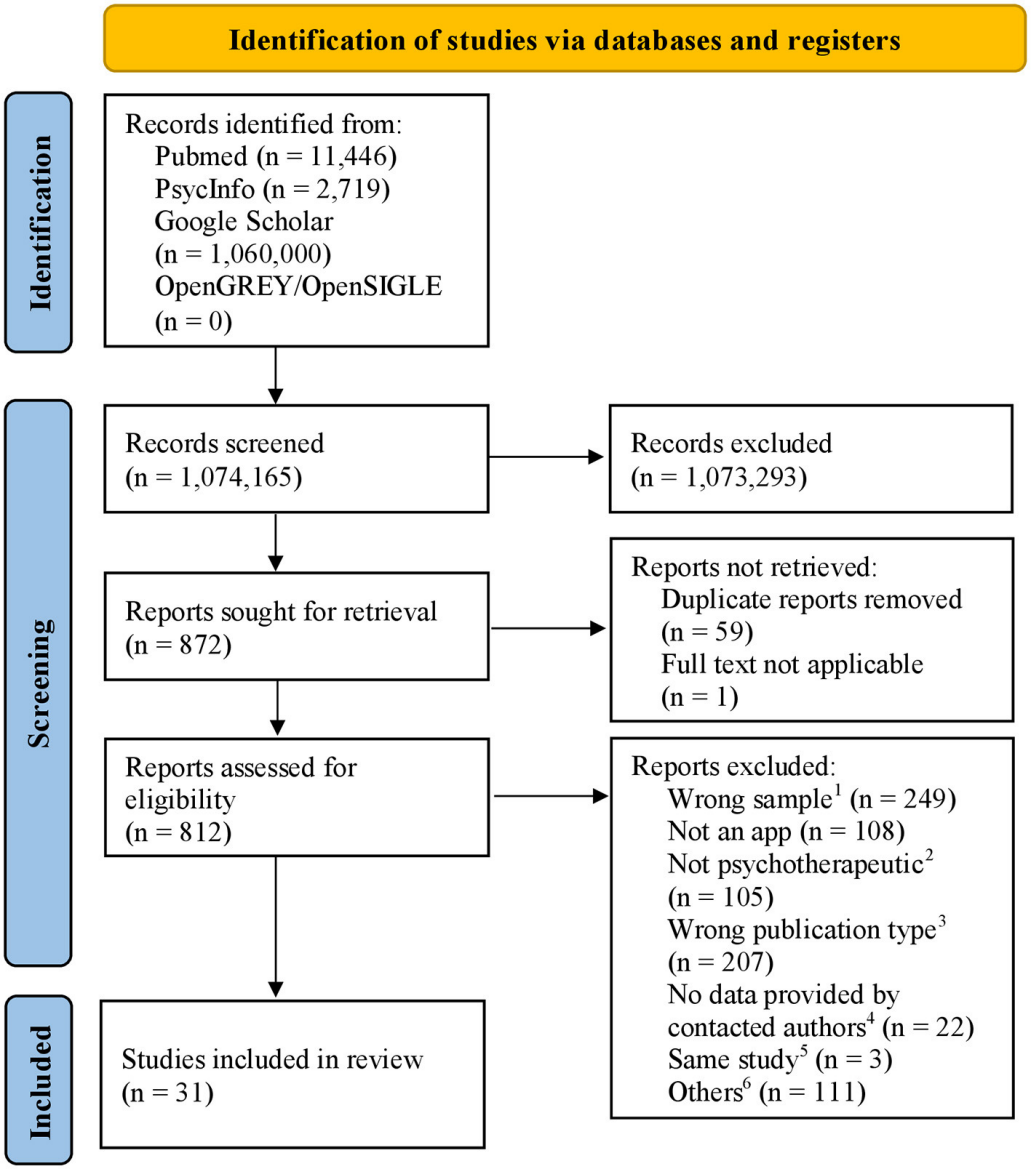
PRISMA flow diagram of the study selection process. ^1^Sample outside the age range, non-clinical sample, adults only, ^2^e.g., management of somatic disease, diagnostic tool, ^3^reviewes, protocols, letter to the editor, ^4^author contacted for providing data of an eligible subsample, because the examined age range was not within the age range of the systematic review, ^5^studies about secondary analyses of other included studies not providing further information for app evaluation, ^6^other reasons, e.g., wrong publication date, studies not about app evaluation.

The search yielded 31 studies fulfilling all inclusion criteria (see Figure 1 for the number of papers included at each stage of the review). Four reviewers were involved in the study selection process and applied eligibility criteria for sample identification. Studies were identified in two steps. First, one reviewer screened titles and abstracts of each of the chosen databases for eligibility. Articles that did not meet the inclusion criteria were excluded. Second, two reviewers screened the full texts of potentially eligible articles. If the two reviewers' assessment of an article was discordant, the disagreement was discussed until a consensus was reached, involving a third party if necessary. The data collection and selection process was managed using the free software rayyan.ai (43) and specifically developed Excel spreadsheets for documentation. We contacted authors of study protocols or studies with samples that were not completely within the age range with a maximum of three email attempts to ask for data provision of sub-samples. Included studies were transferred to a table that presented all key information of the studies (data items): bibliographical data (e.g., authors, contact details of the corresponding author, publication year), app information (name of the app, short description of the app), sample characteristics, trial methods (e.g., study design, type of comparison group), evaluation methods, and outcome data. Finally, two reviewers assessed the quality of each study and their ratings were compared and discussed.

### 2.4 Quality assessment

To assess the quality of each study, the Systematic Assessment of Quality in Observational Research [SAQOR; (44)] was used. The assessment tool enables a differentiated evaluation of the heterogeneous study designs and methods without being limited to randomized controlled trials (RCT). It rates the quality of studies in six categories: sample, control/comparison group, quality of measurement(s) and outcome(s), follow-up, distorting influences, and reporting data. Each category consists of three to five items. Each category is rated as “adequate,” “inadequate,” “unclear,” or “not applicable” according to the ratings of each item. A final quality rating (high, moderate, low, very low) is determined based on the assessment of the six categories. Adapting the tool to the psychotherapeutic context, the items of the category “distorting influences” were summarized in one item asking for potential confounders in general instead of differentiating between two potential key confounders and additional possible confounders mentioned in the article. Two independent researchers carried out the quality assessment. Disagreements in the category ratings were discussed, involving a third party. Interrater reliability between the two reviewers was calculated using Cohen's Kappa (45).

### 2.5 Data extraction and synthesis

To synthesize data of included studies we followed the Synthesis Without Meta-analysis (SWiM) guidelines (46). First, in order to address the high degree of heterogeneity in the methodology and data of evaluation studies, we grouped the included studies by potential outcome measures: (1) qualitative measures only, (2) feasibility measures, (3) measures of app quality, or (4) measures of effectiveness. For studies that only include qualitative outcome measures or examine feasibility or app quality, data were synthesized in the form of a narrative summary. Such studies were grouped under the category “feasibility studies”. Results of studies including effectiveness measures were analyzed separately in two subcategories: effectiveness studies and feasibility studies with (preliminary) effectiveness outcomes. The reported effect estimates were categorized as indicating benefit or harm based on the observed direction of effect of the main effectiveness outcome. Studies that reported no effect of the app intervention were also rated as harmful. For synthesis, votes based on the direction of effects were counted to report the percentage of studies favoring app-based interventions for adolescent psychotherapy context (47). To test if the vote counting results are a statistically significant indicator of app-based intervention being truly effective, a binomial probability test was calculated (48). Differences in effectiveness according to different disorders or areas of application were reported in a narrative synthesis.

## 3 Results

The search identified 31 relevant studies in which 27 different apps were examined. Four of these apps were each addressed in two separate studies. Nine articles were primary effectiveness studies and 19 focused on feasibility measures as primary outcome, of which 14 studies also examined preliminary effectiveness outcomes. Three studies reported qualitative data only. None of the included studies focused on app quality as a primary outcome, but one study examined app quality as a secondary outcome (49). Following the World Bank's definition of high-income economy (50), studies were predominantly conducted in high-income countries (30/31, 97%), with the United States having the highest number of studies (10/31, 32%). Only one study was conducted in a lower-middle income country (India). Most of the evaluated apps focused on specific symptoms or disorders (*n* = 23), with only four acting as transdiagnostic interventions. Overall, 15 apps were based on cognitive behavioral therapy (CBT). Of the 27 apps, 17 were used as stand-alone interventions, seven as therapy-accompanying and three apps were community-network apps, focusing on peer-to-peer treatment or parent-child interactions. Tables 1–3 provide detailed descriptions of the included studies. An overview of the app characteristics is presented in Table 4.

**Table 1:** Study characteristics of each study with primary effectiveness outcomes.

**References, country**	**App**	**Study design**	**Study registration**	**Sample**		**Primary outcome (effectiveness)**					**Quality assessment (SAQOR)**

				**Size**	**Age in years (mean)**	**Measured effectiveness outcome**	**Standardized questionnaires**	**Analysis**	**Vote count**	**Effect estimates**	
Badesha et al. (51), UK	Sanvello	Mixed-methods, single-case experimental design	No	5	15–17 (16.2)	Psychological distress	K-10	Visual analysis on single case level	0	n.a.	3
Dubad et al. (52), UK	Catch It	Mixed-methods, quasi-experimental cohort study	No	47	16–24 (20.7)	Difficulties in emotion regulation	DERS-SF	Mixed Analysis of Variance (ANOVA)	0	n.s.	3
Hilt et al. (53), USA	CARE^a^	RCT	Yes	152	12–15 (13.7)	Trait rumination	CRSQ	Multi-level models	1	*d* = 0.24–0.43	1
Kruzan et al. (54), USA	TalkLife	RCT	Yes	131	16–25 (20.3)	Non-suicidal self-injury	NSSI-AT	Linear mixed models	1	η^2^ = 0.02	1
Li et al. (55), Australia	SleepNinja	Quasi-experimental cohort study	No	49	12–16 (14.1)	Insomnia symptom severity	ISI, PSQI	Hierarchical linear mixed models	1	n.a.	2
Werner-Seidler et al. (56), Australia	SleepNinja	RCT	Yes	264	12–16 (14.7)	Insomnia and depression symptoms	ISI, PHQ-A	Mixed-model repeated measures	1	*d* = 0.28–0.39	1
Rempel et al. (57), Germany	7mind	RCT	No	56	12–19 (15.7)	Obsessive compulsive disorder symptom severity	CY-BOCS	Mixed-effects repeated measures ANOVAS	0	n.s.	3
Schaeffer et al. (58), USA	iKinnect	RCT	Yes	72	13–18 (14.7)	Externalizing behaviors	GAIN-Q3, SRD, CBCL, YSR, ASEBA	Latent growth curve modeling	1	*d* = 0.54–0.84	2
Yang et al. (59), Korea	HARU ASD	RCT	No	26	15–27 (19.3)	Anxiety level	STAI	Mann-Whitney-*U*-Test	1	*r* = 0.52	3

a Same app as Hilt et al. (60). Vote count: 1 = beneficial/0 = harmful or no change. K-10, Kessler Psychological Distress Scale; n.a., not applicable; DERS-SF, Difficulties in Emotion Regulation Scale—Short Form; ns, results were not significant; CRSQ, Children's Response Styles Questionnaire; NSSI, Nonsuicidal Self-Injury Assessment Tool; ISI, Insomnia Severity Index; PSQI, Pittsburgh Sleep Quality Index; PHQ-A, Patient-Health Questionnaire-Adolescent Version; CY-BOCS, German Children's Yale-Brown Obsessive Compulsive Scale; GAIN-Q3, Global Appraisal of Individual Needs; SRD, Self-Report of Delinquency; CBCL, Externalizing behavior scale of the Child Behavior Checklist; YSR, Externalizing behavior scale of the Youth Self Report; ASEBA, Achenbach System of Empirically Based Assessment; STAI, State-Trait Anxiety Inventory.

**Table 2:** Study characteristics of each study with primary feasibility outcomes and secondary preliminary effectiveness outcomes.

**References, Country**	**App**	**Study design**	**Study regis-tration**	**Sample**		**Primary outcome (feasibility)**		**Secondary outcome (preliminary effectiveness)**				**Quality assessment (SAQOR)**
				**Size**	**Age in years (mean)**	**Assessment**	**Qual. int**.^a^	**Measured preliminary effectiveness outcome**	**Standardized questionnaires**	**Vote count**	**Effect estimates**	
Carmona et al. (61), Canada	Doze	Mixed-methods, observational one-group cohort study	No	83	15–24 (n.a.)	TEM, usage data, self-developed	Yes	Sleep Parameters	ISI, CSM, FSS, CESDR-10, STICSA, SF-36	1	*d* = 0.19–0.90	2
Coughlin et al. (62), USA	MiSARA	Mixed-methods, observational one-group cohort study	No	39	16–24 (n.a.)	Self-developed	No	Substance use	AUDIT-C	1	n.a.	3
Geirhos et al. (63), Germany	YouthCoach_CD_	RCT	Yes	30	12–21 (16.1)	INEP-On, IUES, WAI-SR CSQ-I, usage data, self-developed	Yes	Depressive and anxiety symptom severity	PHQ-ADS	0	*d* = 0.30	2
Gonsalves et al. (64), India	POD Adventures	Mixed-methods, observational one-group cohort study	No	248	13–19 (15.6)	CSQ, usage data,	Yes	Mental health symptoms, prioritized problems, stress, wellbeing	YTP, SDQ, PSS, SWEMWBS	1	*d* = 0.31–1.47	3
Grasaas et al. (65), Norway	iCanCope with Pain^TM^	RCT	Yes	73	16–19 (14.4)	Usage data	No	Pain, HRQOL, self-efficacy, anxiety and depression	LPQ, KIDSCREEN-52, GSE, HADS	1	n.s.	1
Hilt et al. (60), USA	CARE^b^	Observational one-group cohort-study	No	80	12–15 (14.0)	Usage data, self-developed	No	Repetitive negative thinking, internalizing symptoms	CRSQ, PWSQ-C, CDI, MASC, PSC.	1	$\eta_{\text{p}}^{2}$ = 0.00–0.33	2
Jeong et al. (66), South Korea	Brake of my Mind (BoMM)	Observational one-group cohort-study	No	3	15–19 (n.a.)	Self-developed	No	Attitudes toward suicide attempts, subjective norms, perceived behavioral control, suicide intentions	n.a.	1	n.a.	3
Miklowitz et al. (67), USA	No name	Observational one-group cohort-study	No	22	13–19 (15.4)	Perceived Ease of Use Scale, usage data, self-developed	No	Depression or mania severity	PSR, YMRS, CDRS-R	1	*d* = 1.58^c^	3
Weintraub et al. (68), USA	No name^d^	Mixed-methods, Observational one-group cohort-study	No	31	13–17 (15.1)	Usage data	No	Mood symptoms & psychosocial functioning	CDRS-R, PQ-B	1	$\eta_{\text{p}}^{2}$ = 0.17–0.36	3
Muscara et al. (69), Australia	BeyondNow & BlueIce	Observational one-group cohort-study	No	20	13–18 (15.5)	Usage data	No	Suicide resilience, self-harm	SRI-25	1	*d* = 0.71	3
Nicol et al. (70), USA	W-GenZ	RCT	No	17	13–17 (14.7)	SUS, usage data, self-developed	No	Depression severity	PHQ-A	1	*d* = 0.98	2
Rauschenberg et al. (71), Germany	EMIcompass	Observational one-group cohort-study	No	10	14–24 (20.3)	Usage data, self-developed	No	General psycho-pathology, depression, anxiety and psychotic symptoms	BSI, GSI, GPTS	1	*r* = 0.30–0.65	2
Reininghaus et al. (72), Germany	EMIcompass	RCT	Yes	92	14–25 (21.7)	Self-developed	No	Psychological distress, stress reactivity	K-10	1	n.s.	2
Thabrew et al. (49), New Zealand	Village	Mixed-methods, observational one-group cohort-study	No	26	16–25 (17.7)	uMARS, usage data, self-developed	Yes	Depression symptoms, suicidal ideation, level of functioning	PHQ-A, SIQ, WHODAS(-CY)	1	*d* = 0.40–0.90	3

a Qualitative Interview was done;

b same app as Hilt et al. (53);

c effect estimate of the PSR (effect estimates of the other measurements were not applicable);

d an adapted version of the app from Miklowitz et al. (67).

n.a., information was not applicable; TEM, Treatment Evaluation Questionnaire; ISI, Insomnia Severity Index; CSM, Composite Scale of Morningness; FSS, Fatigue Severity Scale; CESDR-10, Center for Epidemiologic Studies Depression Scale—revised 10 item version for adolescents; STICSA, State-Trait Inventory for Cognitive and Somatic Anxiety; SF-36, RAND 36-item short form health survey; AUDIT-C, Alcohol Use Disorder Identification Test—Consumption; INEP-On, Inventory for Recording Negative Effects of Online Interventions; IUES, Internet-Use Expectancies Scale; WAI-SR, Working Alliance Inventory-Short Revised; CSQ-I, Client Satisfaction Questionnaire adapted to Internet-based Interventions; PHQ-ADS, Patient Health Questionnaire Anxiety and Depression Scale [combined version of the Patient Health Questionnaire (PHQ-9) and the General Anxiety Disorder Scale-7 (GAD-7)]; CSQ, Client Satisfaction Questionnaire; YTP, Youth Top Problems; SDQ, Strength and Difficulties Questionnaire; PSS, Perceived Stress Scale; SWEMWBS, Short Warwick-Edinburgh Mental Wellbeing Scale; HRQOL, Health related quality of life; LPQ, Lübeck Pain-Screening Questionnaire; GSE, General Perceived Self-Efficacy Scale short form; HADS, Hospital Anxiety and Depression Scale Questionnaire; ns, results were not significant; CRSQ, Children's Response Styles Questionnaire; PWSQ-C, Penn State Worry Questionnaire for Children; CDI, Children's Depression Inventory; MASC, Multidimensional Anxiety Scale for Children; PSC, Pediatric Symptom Checklist; PSR, Psychiatric Status Rating; YMRS, Young Mania Rating Scale; CDRS-R, Children's Depression Rating Scale, revised; PQ-B, Prodromal Questionnaire—Brief; SRI-25, The adapted Suicide Resilience Inventory-25; SUS, System Usability Scale; PHQ-A, Patient Health Questionnaire for adolescents; BSI, Brief Symptom Inventory; GSI, Global Severity Index; GPTS, Paranoid Thoughts Scale; K-10, Kessler Psychological Distress Scale; uMARS, User Version of the Mobile App Rating Scale; SIQ, Suicidal Ideation Questionnaire; WHODAS(-CY), World Health Organization Disability Assessment Schedule (WHODAS was used for patients aged >18 years and WHODAS-CY was used for children and adolescents aged < 18 years).

**Table 3:** Study characteristics of each study with primary feasibility outcomes (without outcomes on preliminary effectiveness).

**References, Country**	**App**	**Study design**	**Study registration**	**Sample**		**Primary outcome (feasibility)**		**Quality assessment (SAQOR)**
				**Size**	**Age in years (mean)**	**Assessment**	**Qual. int**.	
Adams et al. (73), USA	Bright Path	Descriptive cross-sectional study	No	20	14–17 (15.6)	Self-developed	Yes	3
Gómez-Restrepo et al. (74), Colombia	DIALOG+	Descriptive cross-sectional study	No	13	15–17 (16.0)	Self-developed	Yes	4
Li et al. (75), Australia	ClearlyMe	Descriptive cross-sectional study	No	36	12–16 (14.9)	Self-developed	Yes	4
Naccache et al. (76), France	No Name	Descriptive cross-sectional study	No	8	12–18 (15.5)	UEQ, self-developed	Yes	4
Newton et al. (77), Canada	MindClimb	Observational 1-group cohort study	No	8	13–18 (14.0)	Adaptation of CSQ, self = developed	Yes	3
O'Grady et al. (78), Ireland	SafePlan	Descriptive cross-sectional study	No	18	14–16 (n.a.)	SUS, self-developed	Yes	4
Patterson Silver Wolf et al. (79), USA	Bridges To Sobriety	Observational case-control study	No	12	13–19 (n.a.)	Usage data, self- developed	Yes	4
Sharma et al. (80), UK	C.A.L.M BD	Observational 1-group cross-sectional study	No	13	14.5–24.4 (n.a.)	SUS, usage data	n.a.	4

QI, Qualitative Interview; UEQ, User Experience Questionnaire; CSQ, Client Satisfaction Questionnaire for adolescents; SUS, System Usability Survey.

**Table 4:** Characteristics of each app.

**App**	**References, Country**	**Stand-alone vs. therapy-accompanying**	**App content**			**Duration & frequency of app use during the evaluation study**	**Primary outcome of the evaluation study**
			**Specific symptom/disorder or transdiagnostic**	**Overview**	**Based on** ^a^		
BeyondNow & BlueIce	Muscara et al. (69), Australia	Therapy-accompanying	Self-harm, suicidal ideation/behavior	Crisis management: safety plan and skill box for NSSI and suicidal ideation	n.a.	6 weeks, self-selected frequency of use	Feasibility
Brake of My Mind (BoMM)	Jeong et al. (66), South Korea	Stand-Alone	Suicidality	Crisis management: safety plan for suicidal ideation	n.a.	n.a.	Feasibility
Bridges to Sobriety	Patterson Silver Wolf et al. (79), USA	Therapy-accompanying	Substance use disorder	Toolbox and serious games for substance use disorder treatment	n.a.	n.a.	Feasibility
Bright Path	Adams et al. (73), USA	Therapy-accompanying	Substance use disorders and mental health comorbidities	Psychoeducational content and serious games and activities focused on substance use and mental health comorbidities for outpatient health treatment	CBT	Presentation of the app without independent app use	Feasibility
C.A.L.M. BD	Sharma et al. (80), UK	Stand-Alone	Bipolar disorder	Self-management for mood regulation	n.a.	90 days, self-selected frequency of use	Feasibility
CARE	Hilt et al. (60), USA	Stand-Alone	Rumination	Mood monitoring and mindfulness exercises	n.a.	3 weeks, using the app 3 times per day	Feasibility
	Hilt et al. (53), USA	Stand-Alone	Rumination	Mood monitoring and mindfulness exercises	n.a.	3 weeks, using the app at least three times per day	Effectiveness
Catch-It	Dubad et al. (52), UK	Stand-Alone	Transdiagnostic	Mood monitoring and cognitive restructuring of thoughts	n.a.	3 weeks, using the app at least two times per day	Effectiveness
ClearlyMe	Li et al. (75), Australia	Stand-Alone	Depression and anxiety	CBT app intervention for anxiety and depressive symptoms	CBT	n.a	Feasibility
DIALOG+	Gómez-Restrepo et al. (74), Colombia	Therapy-accompanying	Anxiety and depression	Accompanying app for the Dialog+ intervention that structures communication between clinician and patient	n.a.	n.a	Feasibility
DOZE	Carmona et al. (61), Canada	Stand-Alone	Sleep problems (e.g. insomnia, daytime sleepiness, delayed phase circadian rhythms; ranging from subclinical to clinical in terms of their severity)	CBT app intervention for sleep problems	CBT	4 weeks, self-selected frequency of use	Feasibility
EMIcompass	Rauschenberg et al. (71), Germany	Therapy-accompanying	Help-seeking individuals with psychotic, depressive or anxiety symptoms	Ecological Momentary Assessment and supply of training exercises according to in-person intervention sessions	CBT	3-weeks, self-selected frequency of use	Feasibility
	Reininghaus et al. (72), Germany	Therapy-accompanying	Transdiagnostic	Ecological Momentary Assessment and supply of training exercises according to in-person intervention sessions	CBT	6 weeks, self-selected frequency of use	Feasibility
HARU ASD	Yang and Chung (59), Korea	Stand-Alone	Anxiety of ASD patients	CBT app intervention to reduce anxiety in persons with ASD	CBT	66 days, using the app once a day	Effectiveness
iCanCope with Pain^TM^	Graasas et al. (65), Norway	Stand-Alone	Persistent pain	Mood and symptom monitoring, goal setting, self-management strategies, and social support	n.a.	8 weeks, self-selected frequency of use	Feasibility
iKinnect	Schaeffer et al. (58), USA	Community-Network	Conduct problems	Support for caregivers in parenting and dealing with the conduct problems of their children	MST	12 weeks, self-selected frequency of use	Effectiveness
MindClimb	Newton et al. (77), Canada	Therapy-accompanying	Anxiety	Ecological momentary interventions	CBT	Using the app over 6–7 group therapy sessions with a self-selected frequency of app use	Feasibility
MiSARA	Coughlin et al. (62), USA	Stand-Alone	Risky drinking behavior	Daily symptom and mood monitoring and just in time adaptive interventions to prevent alcohol use	JTAI	30 days, using the app at least once a day	Feasibility
No name^b^	Miklowitz et al. (67), USA	Therapy-accompanying	Mood disorders	Interrelated app for adolescents, parents and clinicians for family-focused therapy	CBT	Using the app during the family focused therapy with a self-selected frequency of app use	Feasibility
	Weintraub et al. (68), USA^c^	Therapy-accompanying	Mood disorders, psychotic spectrum disorders	Interrelated app for adolescents, parents and clinicians for family-focused therapy	CBT	9 weeks, self-selected frequency of use	Feasibility
No name^b^	Naccache et al. (76), France	Stand-Alone	Anorexia nervosa	Self-help app for managing emotions and behaviors with a focus on weight loss	CBT	n.a	Feasibility
POD Adventures	Gonsalves et al. (64), India	Stand-Alone	Perceived stress	Lay counselor-guided problem-solving intervention	n.a.	2–3 weeks, using the app at least twice per week	Feasibility
SafePlan	O'Grady et al. (78), Ireland	Stand-Alone	Suicidality	Crisis management: safety plan for suicidal ideation	CBT	Presentation of the app without independent app use	Feasibility
Sanvello	Badesha et al. (51), UK	Stand-Alone	Transdiagnostic	Mental health promotion	CBT	5 weeks, using the app at least once a day	Effectiveness
SleepNinja	Li et al. (55), Australia	Stand-Alone	Insomnia	CBT app intervention for sleep problems	CBT	6 weeks, self-selected frequency of use	Feasibility
	Werner-Seidler et al. (56), Australia	Stand-Alone	Insomnia & depression	CBT app intervention for sleep problems	CBT	6 weeks, self-selected frequency of use	Effectiveness
TalkLife	Kruzan et al. (54), USA	Community-Network	NSSI	Peer support app with psychoeducational elements	n.a.	8 weeks, using the app at least 3 times per week	Effectiveness
Village	Thabrew et al. (49), New Zealand	Stand-Alone	Suicidality and NSSI	Communication app for family/friends support in difficult situations	CBT	4 weeks, self-selected frequency of use	Feasibility
W-GenZ	Nicol et al. (70), USA	Stand-Alone	Depression	Chatbot-delivered CBT for emotion regulation skills	CBT	12 weeks, self-selected frequency of use	Feasibility
YouthCoach_CD_	Geirhos et al. (63), Germany	Stand-Alone	Symptoms of anxiety and depression in AYA with chronic medical conditions (CF, JIA, T1D)	CBT app intervention for anxiety and depressive symptoms	CBT	7 weeks, doing one module per week	Feasibility
7mind	Rempel et al. (57), Germany	Stand-Alone	Obsessive-compulsive disorder	Meditation app	CBT	8 weeks, using the app two times per day	Effectiveness

a Theoretical framework on which the app is based.

a Authors did not mention an app name.

a Adapted version of the app of Miklowitz et al. (67).

NSSI, non-suicidal self-injury; n.a.; information not applicable; ASD, autism spectrum disorder; JTAI, just-in-time adaptive interventions; AYA, adolescent and young adult; CF, cystic fibrosis; JIA, juvenile idiopathic arthritis; T1D: type 1 diabetes.

### 3.1 Results of quality appraisal

Of the 31 studies, four were rated as “high” quality, eight as “moderate” quality, thirteen as “low” quality and six as “very low” quality. The two independent raters showed a moderate interrater reliability of κ = 0.59. Ratings of studies examining effectiveness as primary outcome ranged from high to low quality, with three studies rated as “high”, two as “moderate” and four as “low” quality. The results of the quality appraisal for each study are displayed in the summary of findings Tables 1–3.

### 3.2 App intervention concepts

**Figure 2:**
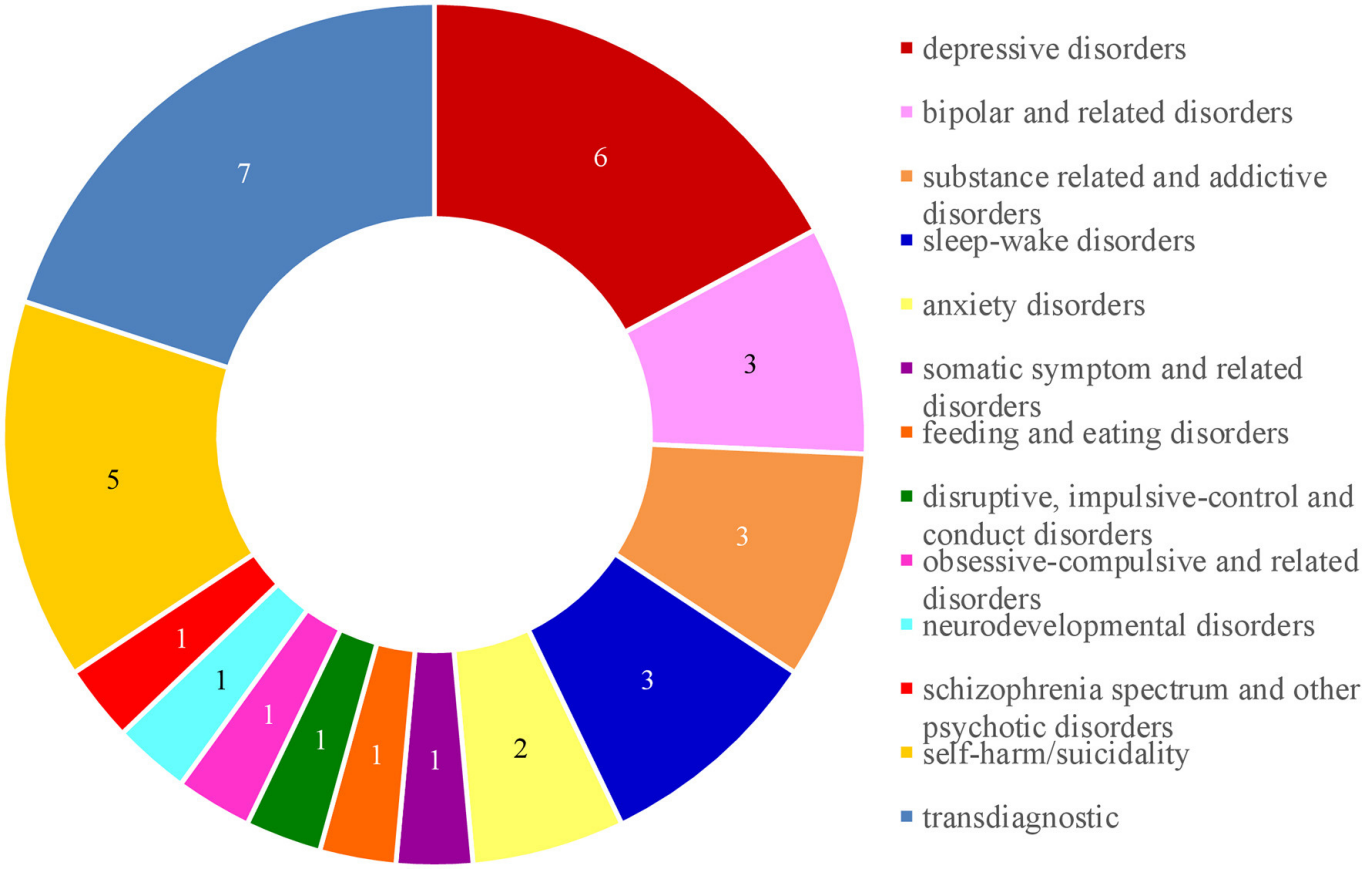
Number of mental health disorder grouped into DSM-5 categories that were addressed in the included apps.

The evaluated mental health apps showed high variety in their areas of application. Most apps were intended as stand-alone, psychological self-help programs. One app was specifically designed to support adolescents during the waiting period for psychotherapy (70). Three out of 27 apps were used as additional treatment to therapy, with two apps designed for specific manualized treatments (67, 68, 74). One app could be used in all standard treatments (52). Furthermore, four apps worked as interrelated apps that connected adolescents with their therapist and primary caregivers (58, 67, 68, 74) or self-selected family members and friends (49). As previously mentioned, four apps were transdiagnostic programs, while 23 apps focused on specific disorders or symptoms. Six out of the 23 apps addressed symptoms across disorders like suicidality and self-harm (*n* = 5) or rumination (*n* = 1). Apps about depressive disorders (*n* = 6) were most common. An overview of disorders addressed by included mental health apps is presented in Figure 2.

### 3.3 App features

Overall, included mental health apps showed a wide range in established features and methods. Five out of the 27 apps followed a defined CBT manual with fixed modules that patients progressed through sequentially (51, 55, 57, 59, 63). Three apps used just-in-time adaptive interventions to treat adolescents (53, 60, 62, 71). All other apps did not specify how the app should be used. Thirty-three percent of apps used gamification elements to motivate patients to use the app (55, 58, 62, 64, 73, 76, 77, 79). Two apps used chatbots in their program to provide patients with personalized treatment (55, 70). Nevertheless, there were also many commonalities between the mental health apps. Fifty-six percent of the mental health apps used mood monitoring as one feature. In total, 63% of apps provided psychoeducational content, with 77% of these apps also providing specific exercises based on the presented psychoeducation. Almost half of the apps aimed to support adolescents in specific behavior changes, with three apps focusing on setting goals (51, 61, 65) and three apps focusing on problem-solving strategies (59, 64, 74). Another frequently used feature was a toolbox with useful skills for difficult situations (*n* = 9). Four out of these nine apps provided a safety plan for suicidal or self-harm crisis management (66, 69, 78, 80). Furthermore, three apps provided a diary feature for app users (59, 61, 78) and four apps had a community forum that enabled app users to communicate with other affected adolescents (49, 54, 65, 75).

### 3.4 Study designs of app evaluations

#### 3.4.1 Studies of effectiveness

Six out of nine effectiveness studies were randomized controlled trials. Two studies were quasi-experimental cohort studies with a pre-post treatment assessment (52, 55) and one study used a single-case experimental design to examine effectiveness of the app intervention (51). Sample sizes ranged from five to 264, with a total of 802 participants across the nine studies. Fifty-six percent of the effectiveness studies examined adolescents under the age of 18 years. Most of the effectiveness studies included a comparison group, except for two studies that did not include any comparison group (51, 55). Five studies used an active control group, of which two studies compared their app-intervention with groups using other similar apps (53, 58) and three studies had a control group in which health related input was provided through other digital technologies (54, 56, 57). Intervention period of app usage lasted between three and 12 weeks with different frequencies of required app usage per day. Most of the apps prescribed daily use (*n* = 7), while two studies required using the app at least three times per day (53, 54). Overall, merely four studies registered their clinical trial and none of the studies published a study protocol.

#### 3.4.2 Feasibility studies with preliminary effectiveness outcomes

As for feasibility studies that also examined (preliminary) effectiveness, 71% of these used an observational one-group cohort study design without a control group. The other four studies conducted a randomized controlled trial, with two studies comparing results with a wait list control group (63, 70), one study using treatment as usual (72) and one study including an attention control group (65). Sample sizes ranged from three to 248, with a total of 774 participants across the 14 studies. Twenty-nine percent of these studies examined adolescents under the age of 18 years. Two studies involved primary caregivers in intervention and assessment, in addition to the adolescent sample (67, 68), and one study involved friends of the participants (49). Intervention periods lasted between 2 and 12 weeks, with two studies requiring daily app usage (60, 62) and three studies requiring app use frequency of at least once a week (63, 64, 68). Of the 14 included feasibility studies with preliminary effectiveness outcomes, three studies registered their clinical trial (63, 65, 72) and one also published a study protocol (72).

#### 3.4.3 Feasibility studies

Five out of eight studies used a descriptive cross-sectional study design to examine feasibility and usability of the apps (73–76, 78). None of the five studies included an intervention period to test the app in real life. For the evaluation component, the app was shown to participants in a single evaluation and assessment session. The other three studies were observational studies, with two studies using the app in regular psychotherapy (77, 79) and one including an intervention period of 90 days as a stand-alone treatment (80). Of the three qualitative studies one included a control group (79). Sample sizes of feasibility studies without effectiveness outcomes and qualitative outcome measures ranged from eight to 36 participants with a mean sample size of 16 adolescents. Moreover, 63% of the studies included mental health professionals or primary caregivers in app evaluation, in addition to the adolescent sample. Overall, a study protocol was published for only one study (74).

### 3.5 Outcomes

#### 3.5.1 Effectiveness outcomes

Considering altogether studies with primary effectiveness outcomes and feasibility studies that examined preliminary effectiveness, 23 studies reported data about effectiveness outcomes, with all studies measuring effectiveness as a reduction of symptoms. Therefore, most studies used standardized questionnaires of symptoms or disorders addressed by the evaluated app. Only one study developed a new questionnaire to measure suicidality (66). An overview of the used outcome measures of each study is presented in Tables 1, 2. There was evidence that mental health apps influenced adolescent psychotherapy, with 19 out of 23 studies favoring the intervention (83%, *p* = 0.002). Four out of 23 studies were judged to be high quality, and all four favored the intervention. Overall, 11 studies were rated low quality, with 73% favoring the intervention. However, looking only at the studies that recorded effectiveness as the primary outcome (*n* = 9), no significant evidence could be found, with six out of nine studies favoring the intervention (67%; *p* = 0.508). Results of vote counting and available effect estimates are presented in the summary of findings tables (Tables 1, 2). Due to the small sample size of the included studies, it was not possible to evaluate effects of mental health apps according to different disorders or areas of application.

#### 3.5.2 Feasibility outcomes

Feasibility was measured with a high heterogeneity in definition and methodology. There was no consistent definition of feasibility aspects across the included studies. Thirteen out of 14 studies used non-validated, self-developed questions about feasibility and acceptability to evaluate their apps. Similarities to standardized questionnaires for feasibility assessment were only found in a few studies: the Client Satisfaction Questionnaire [CSQ; (81)] was used in three app evaluation studies (63, 64, 77), and the System Usability Scale [SUS; (82)] was also used in three app evaluation studies (70, 78, 80). Other validated questionnaires were only used in single studies. As an objective measure, twelve studies presented app usage data as an indicator of feasibility, again showing no consistency in the data categories examined. Furthermore, 11 studies collected qualitative interview data about users' perspectives on the mental health app, with three studies collecting data using solely qualitative methods (74, 75, 79).

## 4 Discussion

### 4.1 Principal findings

The present review gives an overview of studies testing mobile applications in the context of adolescent psychotherapy during the COVID-19 pandemic. In the past 3 years, from June 2020 to June 2023, 31 studies on 27 mental health apps for adolescents between ages 12 and 27 years were published. Table 4 presents an overview of all included mental health apps. Across all studies, effectiveness was defined as a reduction of symptoms and was mostly surveyed with standardized questionnaires about symptoms or disorders. The results of the included studies measuring effectiveness as a primary or secondary outcome indicate that mental health apps are effective for adolescent psychotherapy, with 83% of mental health app studies favoring app-based interventions and the other 17% showing no effect on symptom reduction. No published study showing negative effects on adolescents' wellbeing was found. Nevertheless, these results cannot be presented as evidence of the overall effectiveness of mental health apps for adolescents. Focusing on effectiveness as primary outcome only, we did not find significant evidence that mental health apps are truly effective for adolescents. These findings are consistent with the results of previous research, which also reported promising but inconclusive results of the overall effectiveness of mental health apps (33, 83, 84).

### 4.2 Quality of evaluation studies

One possible reason for the inconclusive results of effectiveness outcomes is the high heterogeneity of study methodology and quality appraisal. Among studies that examined effectiveness as the primary outcome, study quality ranged from low to high, with 44% rated low quality. Two thirds of the effectiveness studies were RCTs, with five studies including active control groups. Two of the high-quality studies used another app within their control group. Three studies did not include comparison groups and one effectiveness study made statements about effectiveness of their treatment using visual analysis of symptom reduction in five participants (51). Another indication of poor research quality in mental health application studies is the non-adherence to established standards, such as good clinical practice guidelines, particularly evident in failure to register their respective studies with a clinical trial registry. Out of the nine effectiveness studies, only four were registered. As other researchers have pointed out, most of the available mental health apps do not provide evidence on their effectiveness (12, 13, 32, 37, 41). In line with the above, we rated a high number of the evaluation studies included in the current review at low quality. Therefore, it remains unclear whether the few apps that show some evidence for their effectiveness were evaluated with studies ensuring good clinical practice and quality.

The other 71% of the included studies focused on feasibility as primary outcome. Considering the steep increase in mental health apps released in app stores (10, 11), the predominance of published feasibility studies over effectiveness studies is not surprising. Following the steps of developing and evaluating new clinical interventions, focusing on feasibility and overall user experience with the new intervention is a common first step before organizing an elaborate effectiveness study. However, Larsen et al. (41) reported that app providers are frequently content with positive results about feasibility and acceptability of mental health apps and do not continue the evaluation of the app further, for example by doing studies on the effectiveness. We also find a high heterogeneity in the quality appraisal and study methodology in the included feasibility studies. Out of the studies measuring feasibility as primary outcome, 68% were rated low or very low quality. Study designs ranged from RCTs to descriptive cross-sectional design studies. Some feasibility studies did not include an intervention period but had single evaluation sessions to rate the developed mental health app. Sample sizes likewise had a wide range from three to 248 included participants. In addition to the high heterogeneity in study design and procedure, measurement of feasibility did not follow a consistent definition. Most of the studies developed own items ranging from questions about having fun using the app or being satisfied with the app, to detailed questions about app functionality or design, to objective usage data like the number of logins or the duration of use. Considering this range of feasibility aspects, it is not possible to make generalized statements about the feasibility of mental health apps for adolescents.

In order to determine the feasibility and effectivity of mental health apps properly, we need researchers to define concepts like app quality and to develop and use corresponding measurements. Future studies should try to apply common scientific standards like study registration, control-group designs, adequate sample size to the field of app evaluation. Multi-method and multi-informand approaches seem promising. However, it is challenging to combine high quality evaluation studies (feasibility studies as well as effectiveness studies) with the fast pace of new developments of mental health apps (14, 38). Nevertheless, this is the only way to expand the knowledge on apps in psychotherapy.

### 4.3 Overview of evaluated mental health apps for adolescents during the COVID-19 pandemic

The area of application of mental health apps did not change significantly during the COVID-19 pandemic. About 70% of the mental health apps were offered as stand-alone treatments, replacing usual psychotherapy services, or being used as a bridge-over during the waiting period for psychotherapy. These results are comparable with previous research, showing that more stand-alone mental health apps were provided than therapy-accompanying mental health apps (34). Most therapy-accompanying apps included in our review provide interrelated app-versions for adolescents and their therapists. All of them were designed for supporting outpatient psychotherapy. Therefore, app features are designed with the aim of supporting patients in therapy homework, monitoring their mood between outpatient sessions or facilitating communication between patient and therapist. Two therapy-accompanying apps did not connect patients directly with their therapists. These apps were designed as accompanying tools for specific manualized treatments with fixed therapy modules following the same order. A new development in this area are apps involving the community network of affected youths. Like Diano et al. (29), former research could typically be divided into two subgroups: stand-alone or therapy-accompanying apps. In the current review, community-network apps were discovered as a third subgroup of the app intervention concept. Three out of the 27 apps were used as community-network apps, connecting youth with primary caregivers or peers. These interventions were predominantly based on family-focused therapy that included primary caregivers or peers as lay counselors and main support for the affected youths. What is remarkable is that two out of the three community-network apps supported the mental health of youth by addressing the issue of self-harm and suicidality. The results of the evaluation studies showed evidence of effectiveness and feasibility without showing negative side effects either on the side of the affected youth or the selected peers or caregivers (49, 54). The development of specific community-network mental health apps could potentially be a consequence of the increased mental health concerns and the resulting pressure to treat during the pandemic. Involving the social environment in supporting the affected youth could relieve the burden on the healthcare system (4, 7, 22) and enable more youth to receive support, especially at an early stage (30). In addition, the feeling of connectedness is an important factor for adolescents (27, 28) and could be another reason for having a greater focus on the social environment in treatment development.

Overall, there is still a greater focus on developing mental health apps for specific disorders or symptoms rather than following a transdiagnostic approach. The current review cannot confirm the trend found by Ellis et al. (9) that transdiagnostic approaches were increasing in the mental health app development. In fact, we found a comparable percentage of transdiagnostic approaches as opposed to those for specific disorders. The increased focus on transdiagnostic treatment approaches is in line with the current psychotherapy research. Instead of developing treatment concepts for clearly defined, specific disorders, psychotherapy research is increasingly trying to develop treatment concepts that focus on symptoms and treatment principles that transcend disorders (85, 86). Moreover, the most common app features, also used in the included disorder-specific mental health apps, were not disorder specific features. For example, mood monitoring and features supporting specific behavior changes were used by almost half of the mental health apps, while skill toolboxes were part of one third of the apps. It can therefore be argued that most of the developed applications exhibited the potential to offer support beyond their primary focus on specific mental health concerns, as each of them incorporated fundamentally transdiagnostic features within their respective platforms. Having a look at the reported disorders of youth, we can see that most adolescents had comorbidities and were diagnosed with more than one specific disorder (18, 87, 88). In order to address the complexity of mental health issues effecting young people, it is necessary to offer transdiagnostic mental health apps. It is not feasible to equip an adolescent with several apps at the time, each one for another psychological problem.

### 4.4 Strengths and limitations

The current review focused on a narrowly defined period from June 2020 to June 2023 in order to examine the pandemic's influence on mental health app development and evaluation. Including only studies published during the COVID-19 pandemic provides a good overview of recently published, studies on mental health apps that possibly take into account the new needs that have arisen in the healthcare sector due to the pandemic. However, the current review did not exclusively include pandemic-related mental health apps, as evaluation studies conducted before the pandemic but published after June 2020 were also included. Another methodological limitation has to be mentioned for the reviewing process. One reviewer for each database, which could introduce selection bias, conducted the initial screening process. Including all kinds of evaluation studies is another strength of the current review. The given overview presents evidence from different evaluation stages and provides a more complete picture of the current developments in the field of mental health apps. However, looking at all types of evaluation studies also weakens the robustness of the results on the effectiveness of mental health apps. When interpreting the results of the included mental health app evaluation studies, we have to consider the overall low quality of study methodology.

### 4.5 Conclusion

Taken altogether, it is evident that within the spectrum of evaluation studies, there are significant disparities in both quality and methodological approaches. There is an urgent need to improve quality of evaluation studies and to ensure that research on mobile mental health complies with the established scientific standards. At the latest since the pandemic, mental health apps have a firm and important role in our healthcare system and are likely to continue to grow in influence in the future. Clinicians as well as adolescents in need are more likely to use digital mental health support, but actual app development policy aggravates the access to high-qualitative evaluated apps. Only with defined standards and high-quality research can we ensure that feasible and effective apps are implemented in psychotherapy with adolescents.

## Data availability statement

The original contributions presented in the study are included in the article/supplementary material, further inquiries can be directed to the corresponding author.

## Author contributions

SW: Conceptualization, Formal analysis, Investigation, Methodology, Visualization, Writing—original draft. KH: Conceptualization, Methodology, Writing—review & editing. MK: Investigation, Writing—review & editing. I-KP: Investigation, Writing—review & editing. TH: Supervision, Writing—review & editing. MS: Funding acquisition, Project administration, Writing—review & editing.
